# Giant Coronary Artery Aneurysm: A Successful Diagnosis

**DOI:** 10.7759/cureus.20429

**Published:** 2021-12-15

**Authors:** Michel El Khoury, Viswajit Reddy Anugu, Chadi Salmane, Boutros Karam, Mohammed Imam, Andrew Warchol

**Affiliations:** 1 Internal Medicine, Northwell Health, New York, USA; 2 Interventional Cardiology, Northwell Health, New York, USA; 3 Cardiology, Northwell Health, New York, USA; 4 Cardiothoracic Surgery, Northwell Health, New York, USA

**Keywords:** coronary artery ectasia (cea), coronary artery bypass graft surgery, coronary artery angiography, coronary aneurysm, coronary artery disease

## Abstract

Coronary artery aneurysms (CAAs) are rare, with giant CAAs being even rarer. The precise pathophysiology of this phenomenon is still unknown. CAAs are seldom reported life-threatening abnormalities of the cardiovascular system. We herein present a case of a 74-year-old man who presented at the hospital complaining of chest pain. An adenosine thallium scan revealed a small, reversible defect in the inferior wall of the left ventricle extending into the apex, consistent with ischemia. Echocardiography uncovered a large right coronary artery (RCA) aneurysm, measuring 5.6 × 7.5 cm. Diagnostic coronary angiography confirmed the presence of a large RCA aneurysm and aneurysmal dilation of the left anterior descending and circumflex arteries with no flow-limiting lesions. A reversed saphenous vein interposition graft was placed from the ascending aorta to the right posterior descending artery. The RCA aneurysmal sac was resected and sent to pathology, which uncovered myxoid degeneration of the media as well as thrombus formation. No complications were encountered during the procedure. Early diagnosis is vital to avoid fatal complications of CAAs, and therapeutic approaches are currently individualized in view of absence of evidence-based management strategies.

## Introduction

Currently, there are no large randomized clinical trials that have compared medical and surgical approaches in treating coronary artery aneurysms (CAAs). Consequently, the knowledge of optimal management of CAAs is based on case reports, small case series, and personal experiences. As such, the therapeutic approach must be individualized and a multidisciplinary team, including interventional cardiologists, cardiothoracic surgeons, and radiologists, must work together to provide the best treatment for their patients. CAAs are defined as a localized dilatation of a coronary artery segment 1.5-fold more than adjacent normal segments. The incidence of CAAs has increased since the dawn of the interventional era. The most common etiologies are atherosclerosis, prior myocardial infarctions, drug-eluting stent placement, connective tissue disorders, vasculitis, some infections, and drugs [1]. CAAs are more prevalent in males, and their incidence differs between ethnicities [2]. CAAs can be asymptomatic or can resemble ischemic heart disease [3]. During childhood, Kawasaki disease, in addition to congenital aneurysms, represents most cases [4]. Giant aneurysms are sometimes misdiagnosed as mediastinal masses or cysts, which can delay the execution of the appropriate treatment plan [5]. This can subsequently lead to fatal complications, such as rupture, fistula formation, or compression of the cardiac chambers. Our discussion will review the possible underlying mechanisms, risk factors, as well as diagnostic modalities, and available therapeutic interventions for CAAs.

## Case presentation

**Figure 1:**
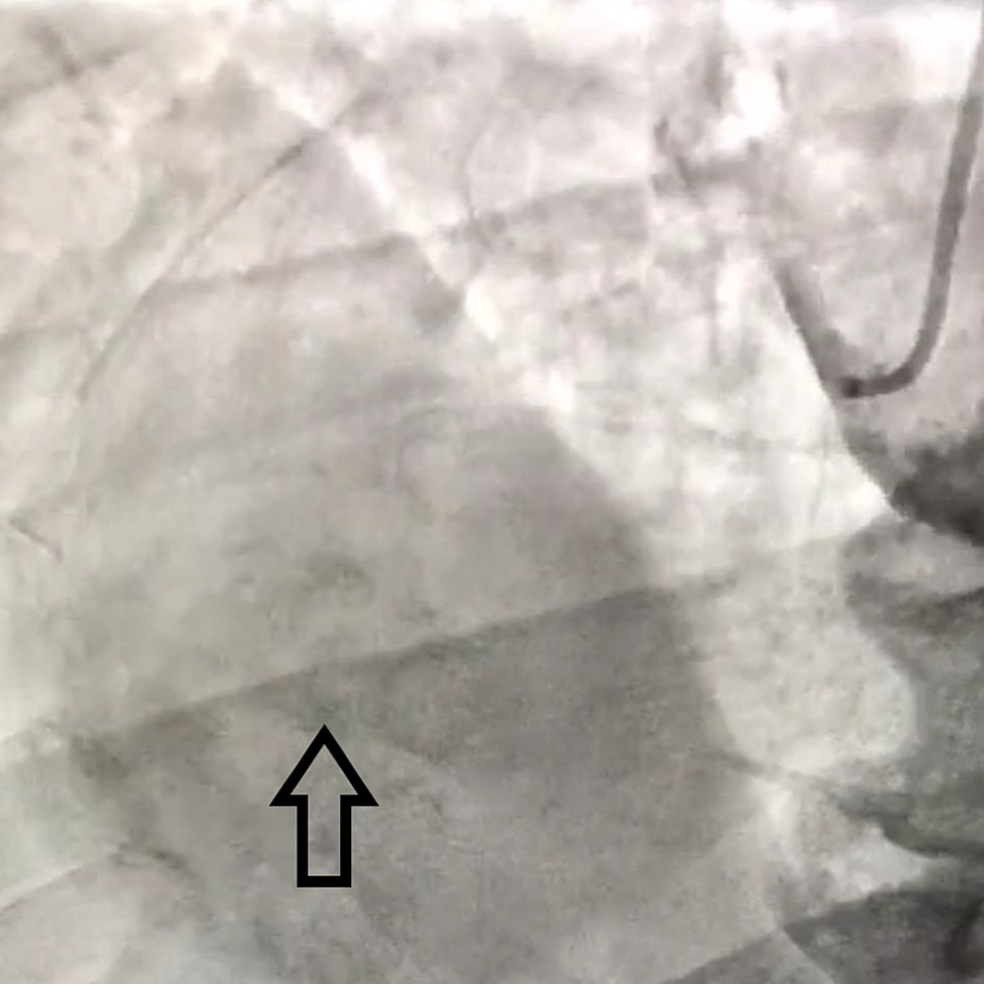
Coronary angiogram showing large right coronary artery aneurysm.

A 74-year-old man presented to the emergency room for chest pain. The pain was retrosternal, pressure-like, of moderate intensity, and started upon awakening from sleep. His past medical history was significant for inferior wall myocardial infarction with right coronary artery (RCA) thrombus and aneurysm formation status post intracoronary urokinase and ReoPro® (abciximab) medication in 1997, hypertension, hyperlipidemia, and prostate cancer. Past surgical history was significant for inguinal hernia repair, cholecystectomy, and appendectomy. Family history was significant for high blood pressure in his father. The patient was a non-smoker, an occasional alcohol consumer, and denied any illicit drug use. Home medications included aspirin, Coumadin, atenolol, simvastatin, paroxetine, and fenofibrate. At presentation, he was in mild distress and complained of typical chest pain. On physical examination, his heart rate was irregular with no audible murmurs and his lungs were clear to auscultation. His heart rate was 56 bpm, and his blood pressure was 110/75 mmHg. Electrocardiography revealed sinus bradycardia with frequent premature ventricular contractions with no signs of infarction or ischemia. Serial troponins were negative. The patient was advised to be admitted for a nuclear stress test and overnight observation but opted to follow up with his cardiologist as an outpatient. An adenosine thallium scan uncovered a small reversible defect in the inferior wall of the left ventricle extending into the apex, consistent with ischemia superimposed on a chronic scar. Additionally, the scan showed inferior wall hypokinesis and a preserved ejection fraction. Echocardiography revealed a large RCA aneurysm measuring 5.6 × 7.5 cm, partially compressing the right atrium and right ventricle, as well as an ejection fraction of 57% with grade 1 diastolic dysfunction. The patient was referred for left and right cardiac catheterization. Diagnostic coronary angiography uncovered right coronary circulation dominance and confirmed the presence of a large RCA aneurysm with an inability to visualize the distal vessel (Figure 1).

**Figure 2:**
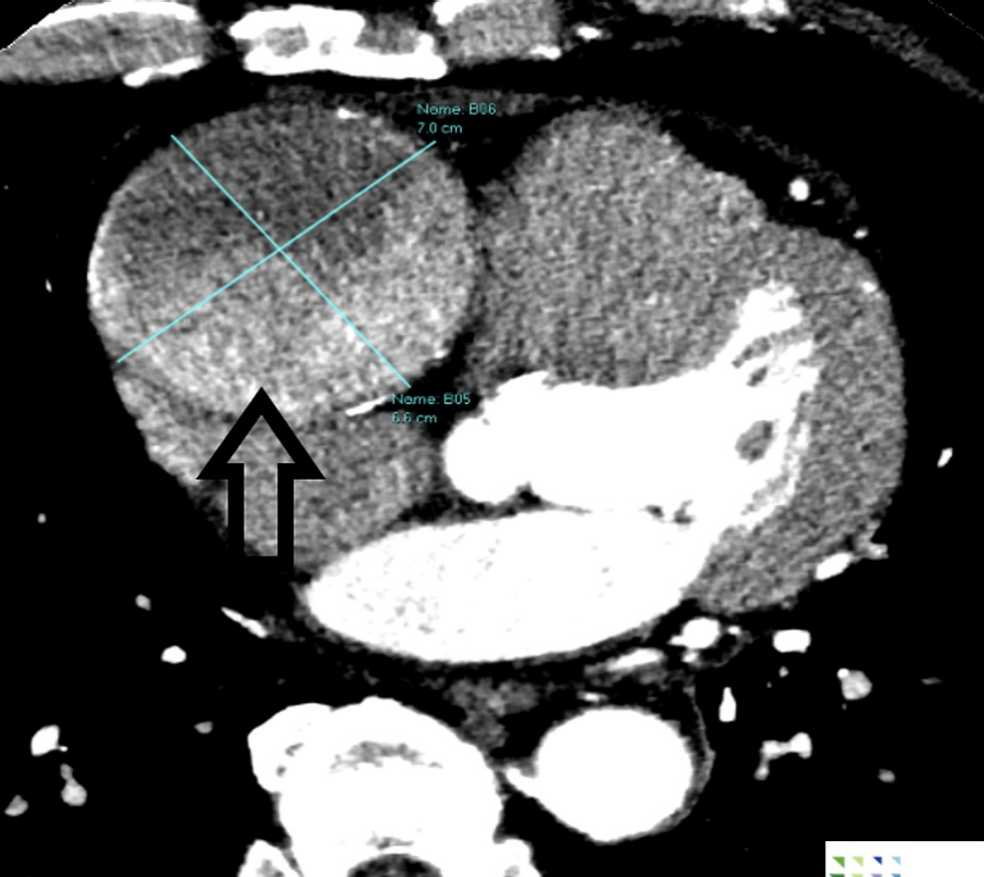
Dual-source CT showing the giant aneurysm (9.0 x 7.5 cm) and the affected vessel.

**Figure 3:**
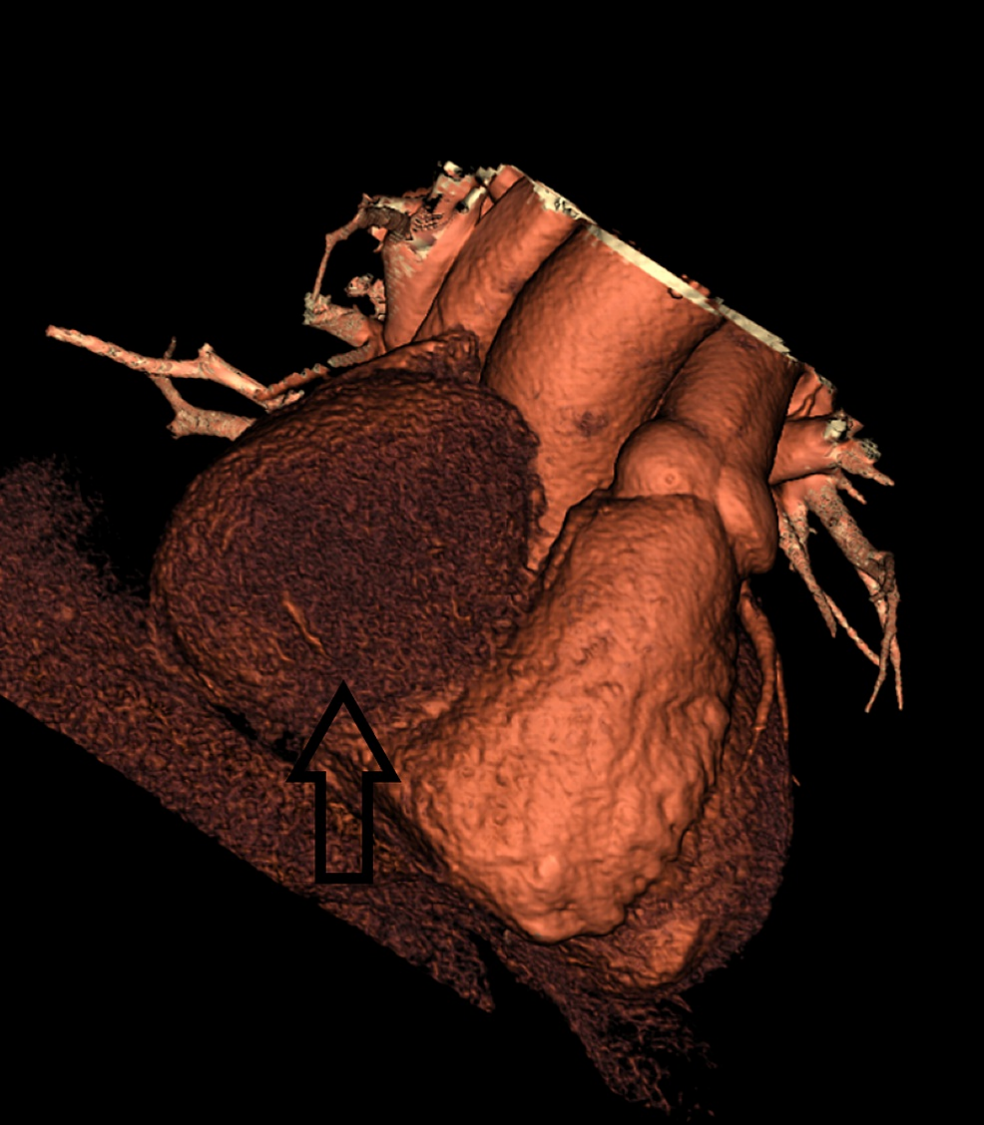
3D reconstructed CT heart with coronaries demonstrating the right coronary artery aneurysm.

It additionally demonstrated aneurysmal dilation of the left anterior descending and circumflex arteries with no flow-limiting lesions. The cardiothoracic team was subsequently consulted. A CT heart scan with coronaries was performed as part of a preoperative workup. Again, the scan revealed a large proximal RCA aneurysm measuring 9 cm in the craniocaudal dimension and 7.5 cm in the transverse dimension. This RCA aneurysm was situated within the atrioventricular (AV) groove and was causing partial compression on the right atrium and ventricle. Proximal to the aneurysm, a patent conus and nodal branches were also present (Figures 2, 3). 

**Figure 4:**
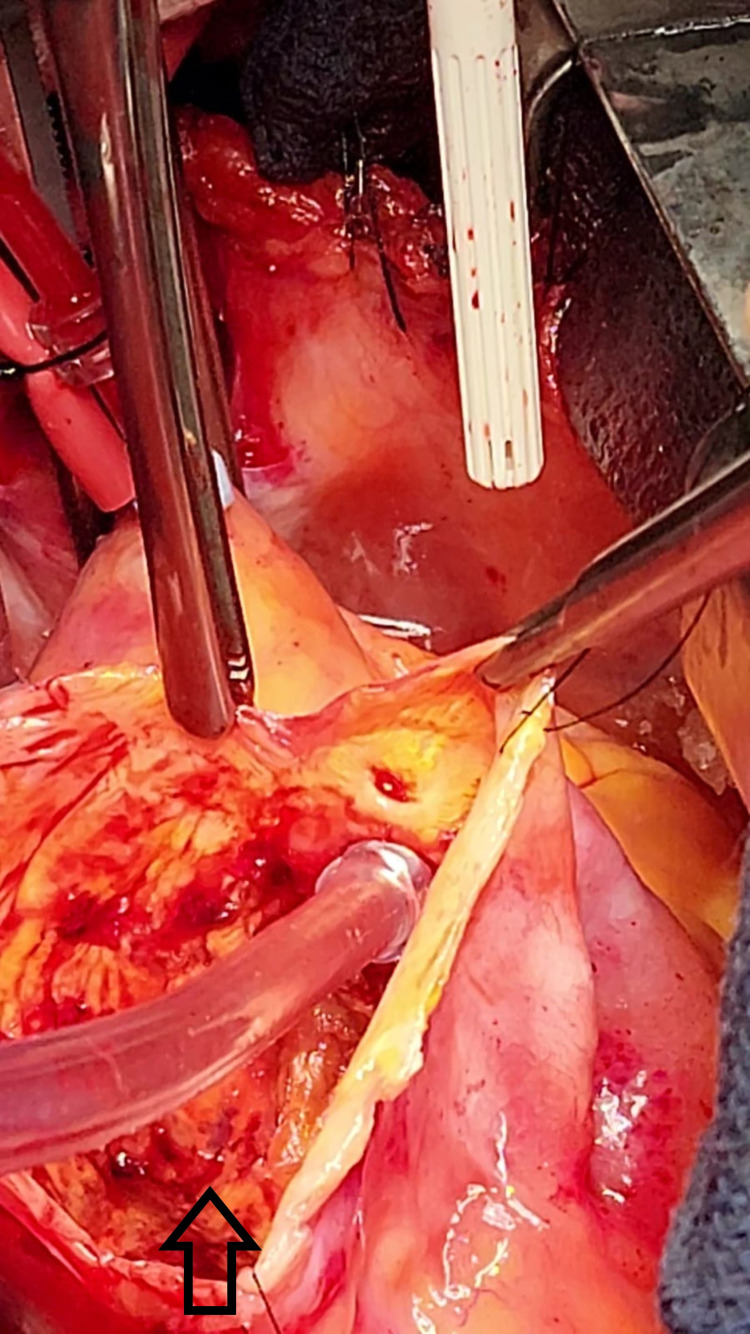
Operative photograph showing the giant aneurysm of the right coronary artery, a view of the inside of the aneurysmal sac, and the resected aneurysm.

A reversed saphenous vein interposition graft was placed from the ascending aorta to the right posterior descending artery. The RCA aneurysmal sac was resected (Figure 4).

Pathology showed myxoid degeneration of the media along with thrombus formation. No immediate complications were encountered during the surgery. The patient was postoperatively kept on vasopressors for 24 hours, extubated, and was discharged on postoperative day five. The postoperative follow-up was uneventful, and the patient was able to regain his functional capacity.

## Discussion

CAAs were first described postmortem by Morgagni in 1761 [6]. CAAs are termed “giant” if their diameter transcends four times the reference vessel diameter or if they are >8 mm in diameter [4]. Coronary dilatations >2 cm are exceedingly rare [7]. RCA disease is the most prevalent coronary artery aneurysm and is involved in 40-70% of CAAs. This is followed by the left anterior descending artery with 35% and the left circumflex artery with 25% of cases. Involvement of multiple coronary arteries or the left main artery is rarer, representing only 3.5% of CAAs. This is usually seen in atherosclerotic or inflammatory CAAs. These CAAs are predominately asymptomatic and are found incidentally on coronary angiography. This diagnostic modality can provide information on the size, shape, and location of the CAA, but it does not reflect information about the vessel wall. This could lead to underestimation of the actual size of the aneurysm or even cause the CAA to be overlooked as it may be occluded by a large thrombus or plaque. For this reason, intravascular ultrasound is used to provide transmural images of the coronary arteries, allowing for information on the arterial wall structure and luminal composition to be derived.

A previously conducted study used intravascular ultrasound to assess CAAs diagnosed by angiography in 77 patients [8]. Most angiographically diagnosed aneurysms had the morphology of complex plaques or normal segments with adjacent stenoses. Only one-third were true or pseudoaneurysms. Other non-invasive tests, such as echocardiography, CT coronary angiogram (CTCA), and magnetic resonance coronary angiography (MRCA), could be used for the follow-up in patients with suspected or treated CAAs. However, with MRCA, the distal vessel cannot be visualized and also carries a lower spatial resolution when compared to CTCA. With regard to a CAA being an exceedingly rare condition, there are currently no large randomized clinical trials that compare different therapeutic management approaches in response to a CAA. Consequently, the knowledge of optimal management is based on case reports, small case series, and personal experiences. The therapeutic approach must be individualized depending on the etiology, location, symptoms, size, progression over time, presence of an infection, and extent of any coexisting atherosclerosis. Medical therapy with statins should be adopted in asymptomatic CAAs with the purpose of blocking the inflammatory pathway responsible for the development of CAAs. In case thrombosis or embolism is a concern, long-term use of antiplatelet or anticoagulants should be considered. Percutaneous coronary intervention with covered stents can be used in patients with suitable anatomy, but the risk of restenosis is high, especially in CAAs >10 mm [9]. For this reason, surgical intervention is recommended in large CAAs at risk of rupture; CAAs with compression of the cardiac chambers; progressive enlargement seen on imaging; evidence of emboli from the aneurysm to the distal coronary bed, resulting in myocardial ischemia; complications such as fistula formation; and CAAs of the left main artery or near the bifurcation of large branches. 

## Conclusions

Giant CAAs are rare. Clinical presentation can vary and maintaining a high index of suspicion is vital in avoiding delayed diagnosis and increased risk for cardiovascular complications. This was secondary to the widespread use of coronary angiography, high-resolution CT, and MRI. However, evidence-based management strategies have yet to be established.

The management of CAAs remains a challenge and should be individualized to each patient based on a comprehensive clinical evaluation. These evaluations should take into consideration the patient’s cardiovascular risk factors, comorbidities, and the nature and anatomy of the lesion. Further studies are required to better understand the exact pathophysiology behind CAAs and to promote prevention. Until then, we advise physicians to address the underlying modifiable risk factors, especially in atherosclerotic CAAs.
